# HIV-1 testing of young febrile adults seeking care for fever in sub-Sahara Africa

**DOI:** 10.1093/inthealth/ihu026

**Published:** 2014-06

**Authors:** Eduard J. Sanders

**Affiliations:** Centre for Geographic Medicine Research – Coast, Kenya Medical Research Institute (KEMRI) – Kilifi, Box 230, Kilifi, Kenya; Nuffield Department of Clinical Medicine, University of Oxford, Headington, UK

**Keywords:** Acute HIV-1 infection, Febrile adult, Sub-Saharan Africa

Individuals with acute HIV-1 infection (AHI) are highly contagious,^1^ and may account for a large number of new HIV-1 infections.^2^ Some individuals with AHI remain asymptomatic, but data from East African cohort studies suggest that most (53–75%) experience an acute ‘malaria-like’ illness approximately 2 weeks after infection.^3–5^ Common symptoms of AHI include fever, joint and muscle pains, headache, fatigue, diarrhoea and sometimes rash.^6^ Despite this common presentation, few clinicians in sub-Saharan Africa (SSA), the region most affected by the HIV-1 epidemic, have been trained to put AHI in the differential diagnosis of the young febrile adult patient seeking care.^7^

While rapid point-of-care RNA or p24 antigen tests are in development,^8^ most clinicians attending to patients in primary care facilities in SSA have little else to offer than a rapid HIV-1 antibody test to rule out prevalent HIV-1 infection. This test should be repeated after 3–4 weeks to rule out HIV-1 antibody seroconversion in HIV-1-seronegative patients. The uptake, however, of provider-initiated testing and counselling (PITC) in SSA has been very low,^9^ and repeat testing is not recommended for febrile patients.^10^

Using RNA testing, Bebell et al. reported in 2010 that 1–3% of adults who sought care for suspected malaria in Uganda actually had acute or early HIV infection.^11^ Similarly, and also in 2010, Serna-Bolea et al. reported that 3.3% of patients who sought care for fever at a district hospital in southern Mozambique had AHI.^12^ A third study conducted in coastal Kenya in 2011 found that most adults seek urgent healthcare when acquiring HIV-1 and are frequently treated for malaria.^4^ Unfortunately, these three studies have not yet led to greater recognition of AHI in young sexually active adult patients presenting with fever in SSA.

Why is AHI rarely diagnosed? In this issue (p. 82–92), Prins and colleagues (including this author) present data from a systematic literature review of studies evaluating adult outpatients presenting with uncomplicated fever in SSA over the past decade.^13^ We systematically documented whether testing for prevalent HIV-1 and AHI was performed; searched for HIV testing recommendations in national and international guidelines currently available for the management of febrile adults; and conducted an audit of current PITC practice and AHI diagnostic capacity at nine health facilities in two adjacent towns in coastal Kenya.

The systematic review identified 43 eligible studies for detailed review. The identified studies investigated the following fever aetiologies: malaria only (34 studies), AHI and malaria (2 studies), human herpes virus-8 and hepatitis B virus (1 study), pneumococcal bacteraemia (1 study), flaviviruses (1 study), influenza virus (1 study), Lassa virus (1 study), intestinal helminths (1 study) and schistosomiasis (1 study). Most (27/43) of the studies were carried out in Eastern and Southern Africa, which bear the brunt of HIV-1 epidemic. In only two studies, those where the primary interest was acute HIV-1 infection, were all study participants tested for HIV-1. One study did very limited testing for prevalent HIV-1 infection. In 13 studies, HIV-1 testing was not done at all. Finally, in 27 studies reporting no HIV testing data, information could not be obtained.^13^

The WHO publication entitled ‘*Acute Care: Integrated Management of Adolescent and Adult Illness (IMAI)*’, first published in 2004 and updated in 2009,^14^ recommends considering HIV-1 related illness in cases of ‘unexplained fever for >30 days’ without a positive malaria smear or dipstick, and does not mention AHI as a possible cause of fever during acute care-seeking. It is therefore not surprising that no national guideline mentioned AHI. The audit of PITC of patients aged 18–29 years seeking care reported in Prins et al. revealed low uptake (16.1%).^13^ Healthcare workers cited stock outages as a reason for low testing rates in government facilities, and fees for testing as a barrier in private facilities.

The review by Prins et al.^13^ paved the way for a prospective study targeting young adult patients for AHI evaluation in coastal Kenya.^15^ In this recently published study, 3602 adults were evaluated for prevalent HIV-1 infection (prevalence: 3.9%). Patients with fever were more likely to have prevalent HIV-1 infection than those without fever (9.1% vs 3.3%, p<0.001). AHI was diagnosed in 5 of 506 HIV-1-negative or HIV-1-discordant patients who met AHI risk criteria and were completely evaluated. Of these 5 AHI cases, 4 were diagnosed among the 241 patients with fever (prevalence 1.7%) versus 1 among 265 non-febrile patients (prevalence 0.4%, p=0.1). Malaria was confirmed by PCR in 4 (1.7%) of the 241 febrile patients.^15^ This paper received an editorial comment, the title of which speaks for itself: ‘Acute HIV-1 infection in SSA: a common occurrence overlooked’.^16^

The systematic review and audit of current practice in this issue (p. 82–92), together with the recent study from coastal Kenya suggesting that AHI is as common as malaria, call for urgent public health action. First, and in support of the recommendation by Prins et al.,^13^ the WHO guidelines need to emphasise the importance of HIV-1 testing in febrile adults, especially in areas with high levels on ongoing HIV-1 transmission. Second, fever aetiology studies need to be conducted in adult outpatients in order to inform local clinicians' understanding of the causes of fever. Third, clinicians should consider AHI in the differential diagnosis of young, sexually active adults seeking urgent care for fever. While the diagnosis of AHI using rapid HIV-1 antibody tests is cumbersome, HIV-1 testing and retesting is currently recommended for patients who present with a STI after their first HIV-1 test was negative.^10^ This recommendation should be extended to include young sexually active adults seeking care for fever. Fourth, rapid point-of-care RNA tests developed for HIV-1 care management should be evaluated in HIV-1 antibody negative patients presenting with fever.^8^ Last, and most importantly for HIV-1 control, febrile patients diagnosed with acute or prevalent HIV-1 should be linked to care and offered antiretroviral treatment according to nationally recommended guidelines. Improved testing and linkage to care for young, febrile adults with HIV-1 infection will not only improve outcomes for patients with undiagnosed prevalent infection but also reduce HIV-1 transmission, especially if patients with AHI can be identified, offered immediate ART and supported to adhere to treatment.^17,18^

## References

[1] Hollingsworth TD, Anderson RM, Fraser C (2008). HIV-1 transmission, by stage of infection. J Infect Dis.

[2] Miller WC, Rosenberg NE, Rutstein SE, Powers KA (2010). Role of acute and early HIV infection in the sexual transmission of HIV. Curr Opin HIV AIDS.

[3] Lindback S, Thorstensson R, Karlsson AC (2000). Diagnosis of primary HIV-1 infection and duration of follow-up after HIV exposure. Karolinska Institute Primary HIV Infection Study Group. AIDS.

[4] Sanders EJ, Wahome E, Mwangome M (2011). Most adults seek urgent healthcare when acquiring HIV-1 and are frequently treated for malaria in coastal Kenya. AIDS.

[5] Lavreys L, Thompson ML, Martin HL (2000). Primary human immunodeficiency virus type 1 infection: clinical manifestations among women in Mombasa, Kenya. Clin Infect Dis.

[6] Cooper DA, Gold J, Maclean P (1985). Acute AIDS retrovirus infection. Definition of a clinical illness associated with seroconversion. Lancet.

[7] Crump JA, Gove S, Parry CM (2011). Management of adolescents and adults with febrile illness in resource limited areas. BMJ.

[8] Schito M, Peter TF, Cavanaugh S (2012). Opportunities and challenges for cost-efficient implementation of new point-of-care diagnostics for HIV and tuberculosis. J Infect Dis.

[9] Roura M, Watson-Jones D, Kahawita TM (2013). Provider-initiated testing and counselling programmes in sub-Saharan Africa: a systematic review of their operational implementation. AIDS.

[10] WHO (2010). Delivering HIV test results and messages for re-testing and counselling of adults.

[11] Bebell LM, Pilcher CD, Dorsey G (2010). Acute HIV-1 infection is highly prevalent in Ugandan adults with suspected malaria. AIDS.

[12] Serna-Bolea C, Munoz J, Almeida JM (2010). High prevalence of symptomatic acute HIV infection in an outpatient ward in southern Mozambique: identification and follow-up. AIDS.

[13] Prins HAB, Mugo P, Wahome P (2014). Diagnosing acute and prevalent HIV-1 infection in young African adults seeking care for fever: a systematic review and audit of current practice. Int Health.

[14] WHO. Acute Care (2009). Integrated Management of Adolescent and Adult Illness (IMAI).

[15] Sanders EJ, Mugo P, Prins HA (2014). Acute HIV-1 infection is as common as malaria in young febrile adults seeking care in coastal Kenya. AIDS.

[16] Powers KA, Cohen MS (2014). Acute HIV-1 infection in sub-Saharan Africa: A common occurrence overlooked. AIDS.

[17] Cohen MS, Smith MK, Muessig KE (2013). Antiretroviral treatment of HIV-1 prevents transmission of HIV-1: where do we go from here?. Lancet.

[18] Le T, Wright EJ, Smith DM (2013). Enhanced CD4+ T-cell recovery with earlier HIV-1 antiretroviral therapy. N Engl J Med.

